# Supplementary material for: Global projections of future urban land expansion under shared socioeconomic pathways
Source: Nat Commun. 2020 Jan 27;11:537. doi: 10.1038/s41467-020-14386-x (PMC6985221; doi:10.1038/s41467-020-14386-x)
Supplement: Supplementary file 2 — Supplementary Information File [file 41467_2020_14386_MOESM2_ESM.pdf]

Supplementary Information

Global Projections of Future Urban Land Expansion under Shared Socioeconomic Pathways

Chen et al.

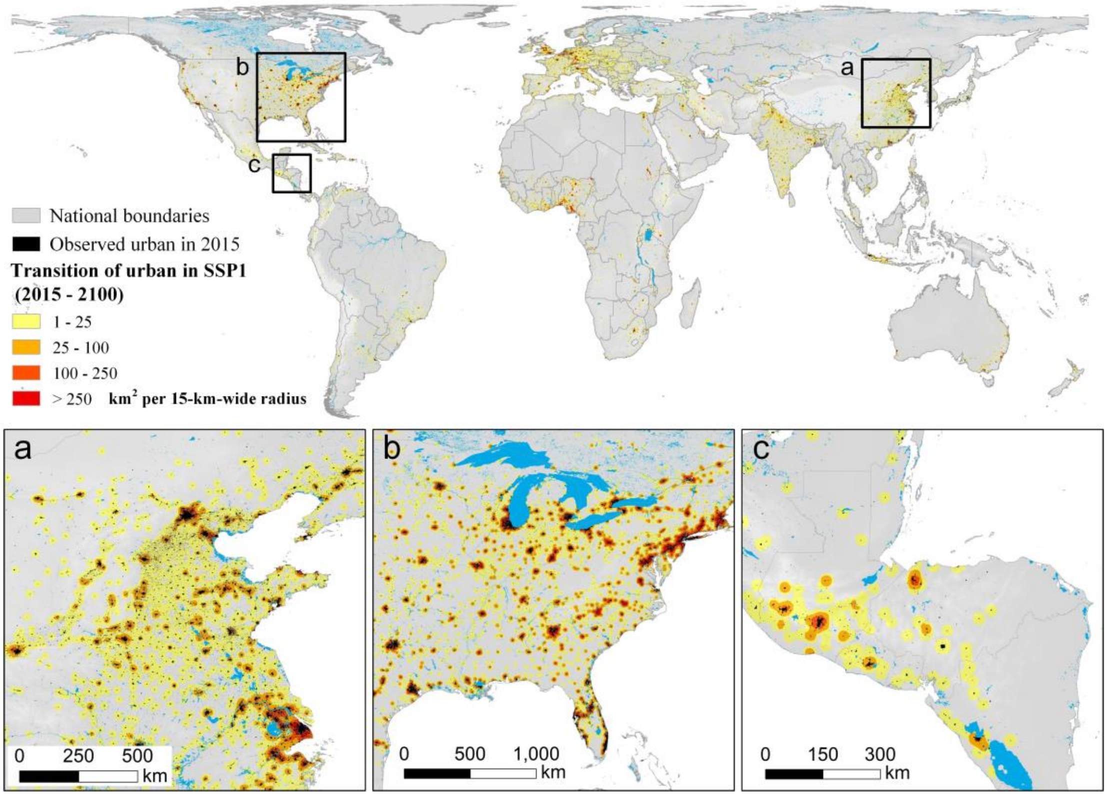

Supplementary Figure 1 | Simulation results in 2100 in SSP1. a) eastern China; b) eastern USA; c) LAM-L. Focal statistics are used as an instrument for better visualization.

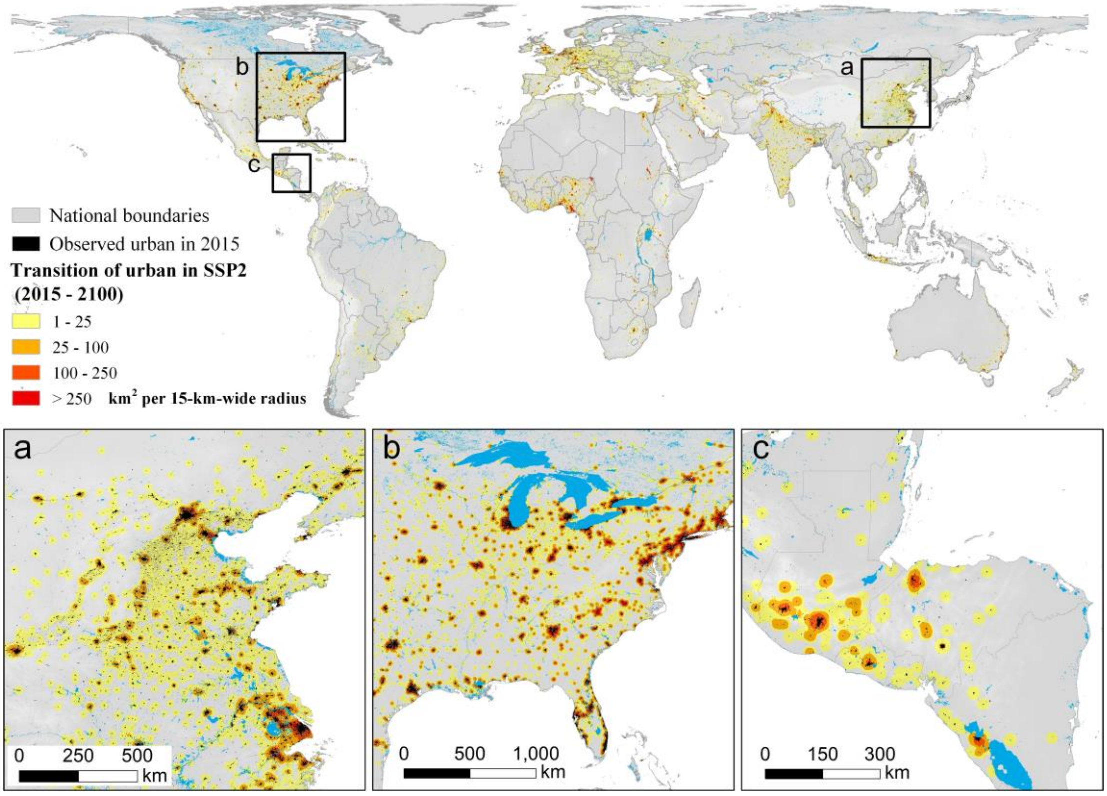

Supplementary Figure 2 | Simulation results in 2100 in SSP2. a) eastern China; b) eastern USA; c) LAM-L. Focal statistics are used as an instrument for better visualization.

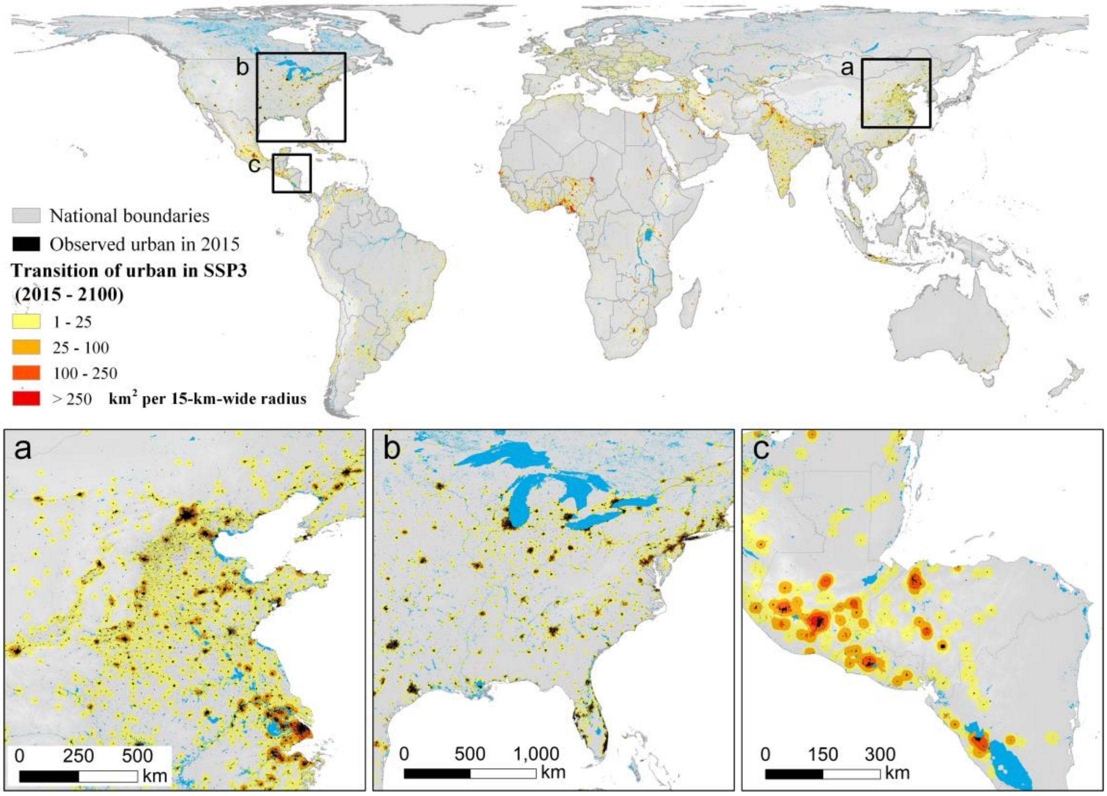

Supplementary Figure 3 | Simulation results in 2100 in SSP3. a) eastern China; b) eastern USA; c) LAM-L. Focal statistics are used as an instrument for better visualization.

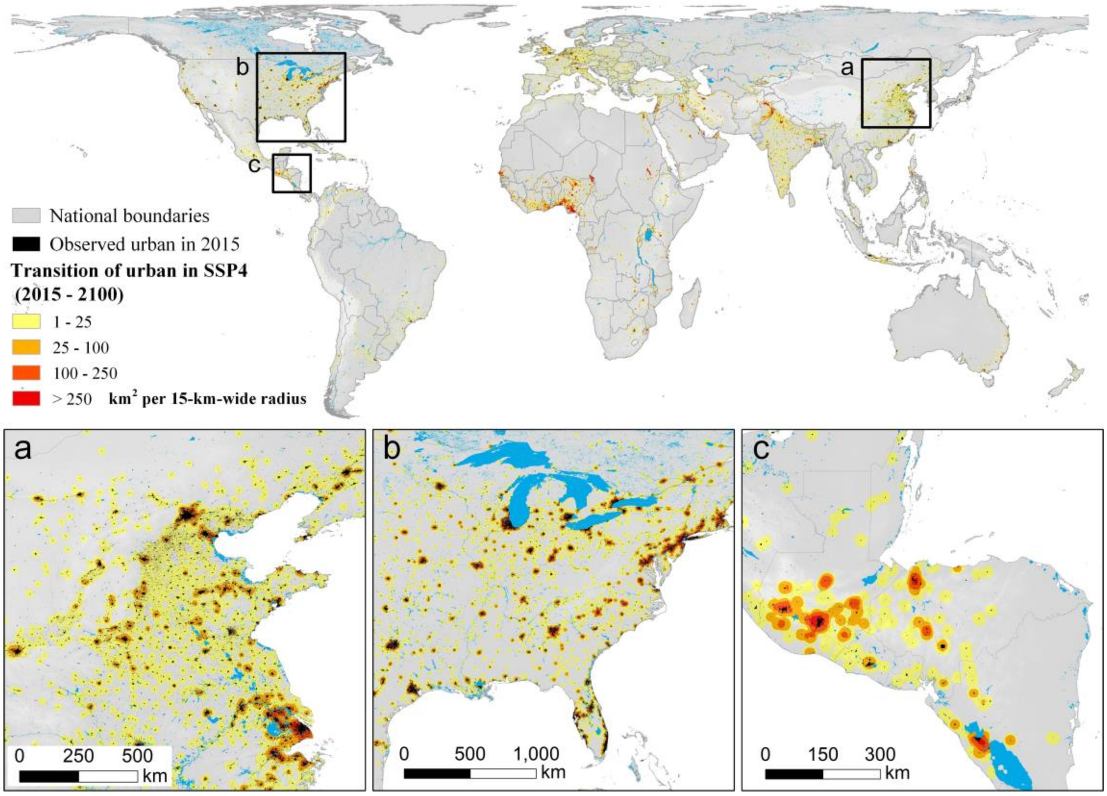

Supplementary Figure 4 | Simulation results in 2100 in SSP4. a) eastern China; b) eastern USA; c) LAM-L. Focal statistics are used as an instrument for better visualization.

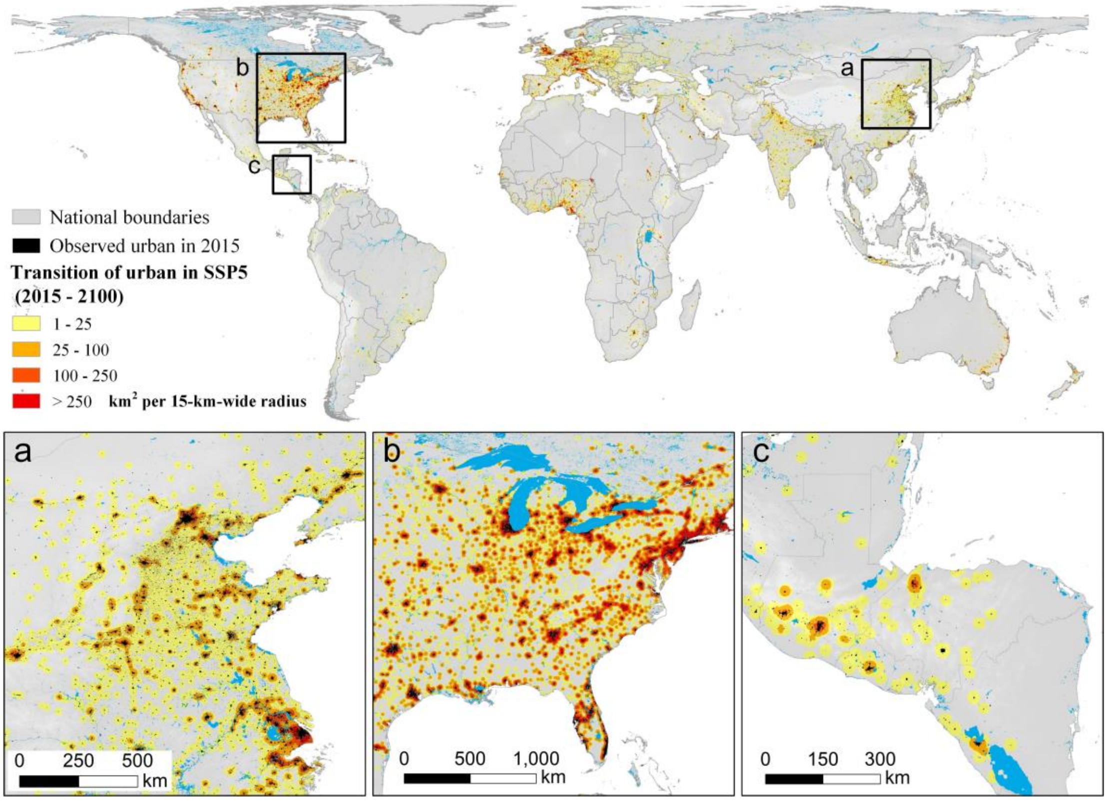

Supplementary Figure 5 | Simulation results in 2100 in SSP5. a) eastern China; b) eastern USA; c) LAM-L. Focal statistics are used as an instrument for better visualization.

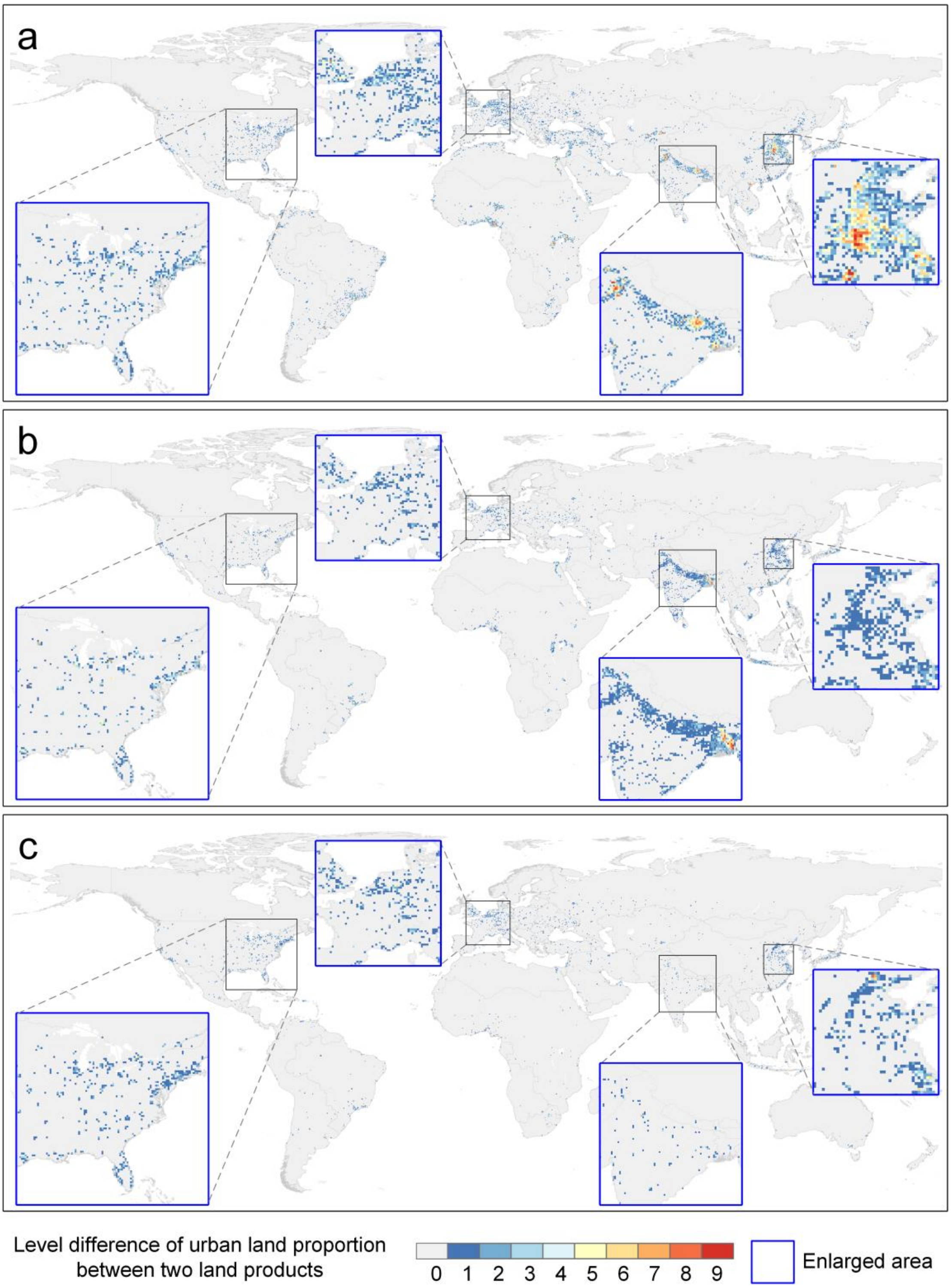

Supplementary Figure 6 | **The absolute difference between our product and other global urban land products in 2030. (a) Ours in SSP2 and Seto's; (b) Ours in SSP2 and Zhou's; (c) Ours and LUH2's in SSP2.** The comparisons are based on the urban land proportions in a 0.25 degree grid. The level are divided by 10% of the difference in the urban land proportions. That is, "Level

0" means the difference in the urban land proportion between two products is less than 10%, "Level 1" means less than 20%, and so on.

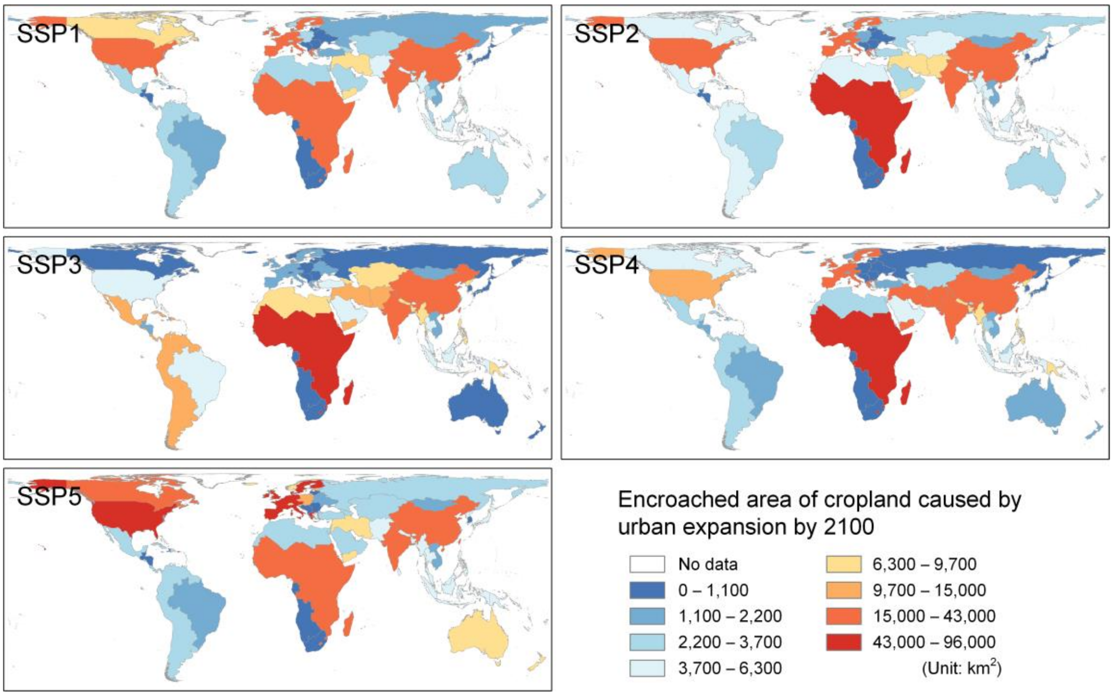

Supplementary Figure 7 | Encroached cropland area caused by urban expansion in each SSP scenario by 2100.

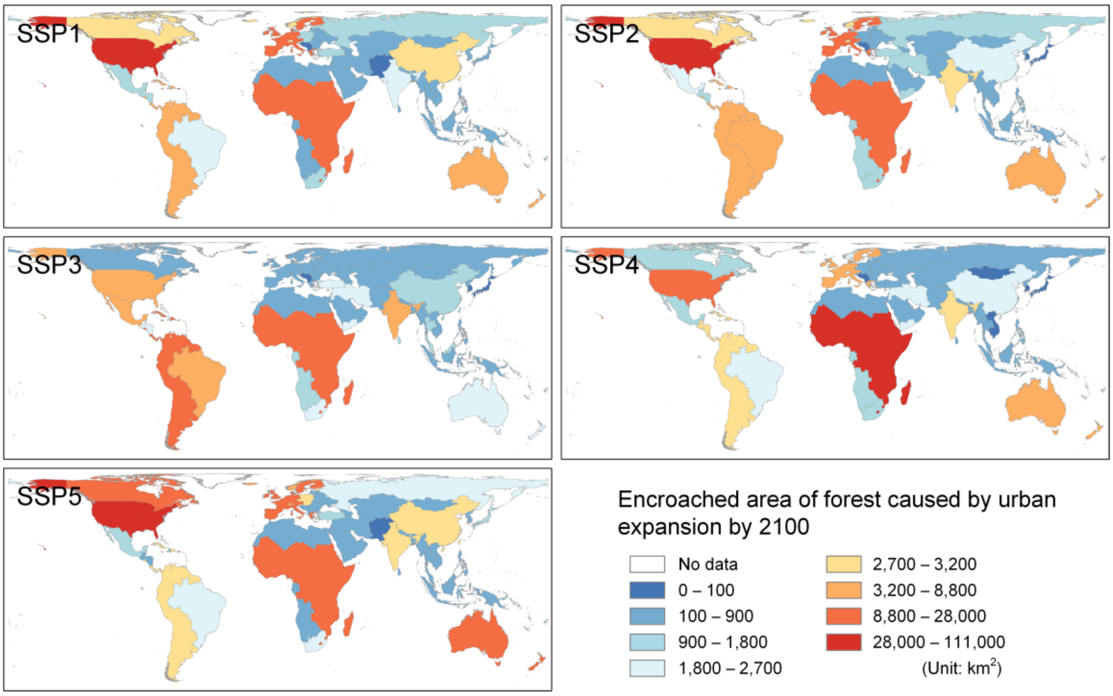

Supplementary Figure 8 | Encroached forest area caused by urban expansion in each SSP scenario by 2100.

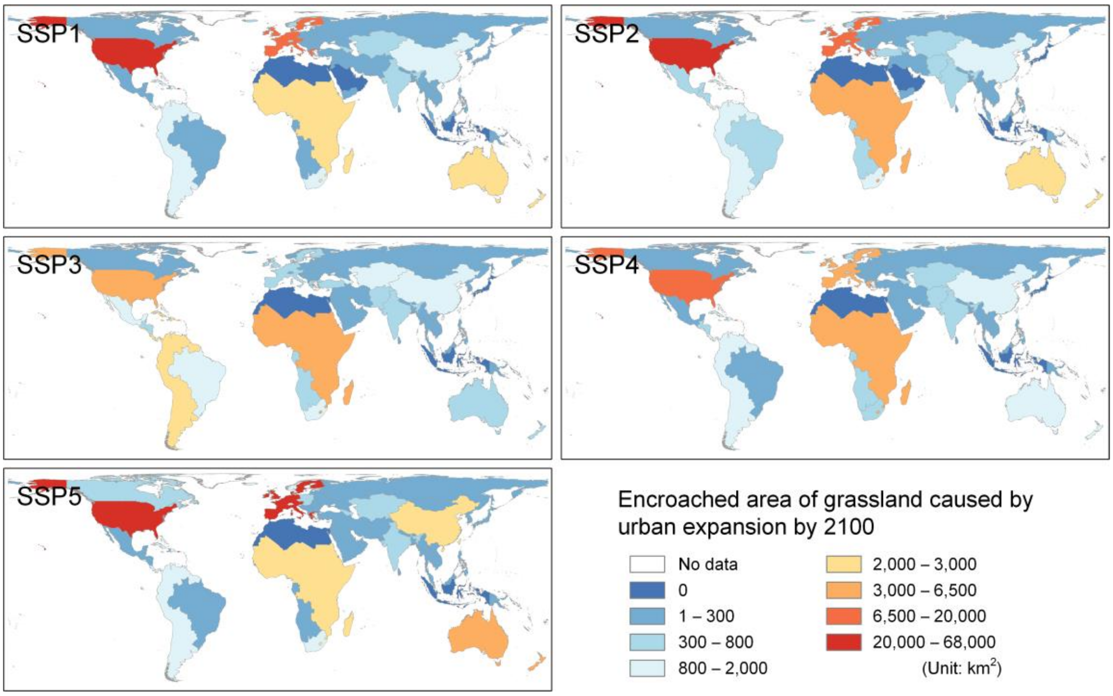

Supplementary Figure 9 | Encroached grassland area caused by urban expansion in each SSP scenario by 2100.

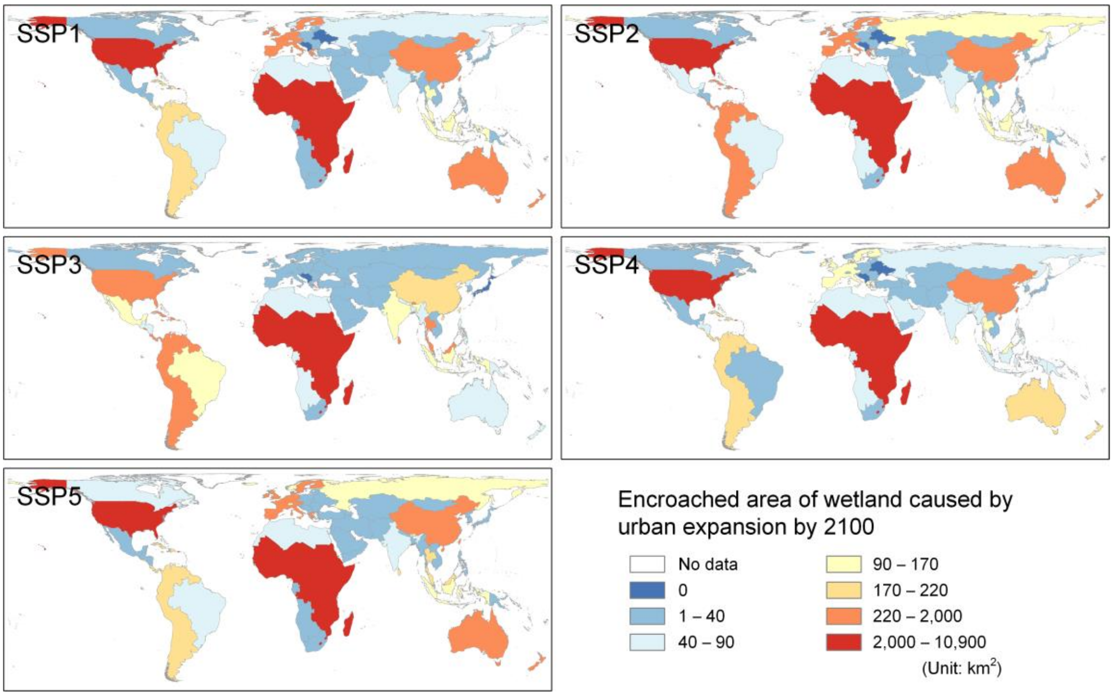

Supplementary Figure 10 | Encroached wetland area caused by urban expansion in each SSP scenario by 2100.

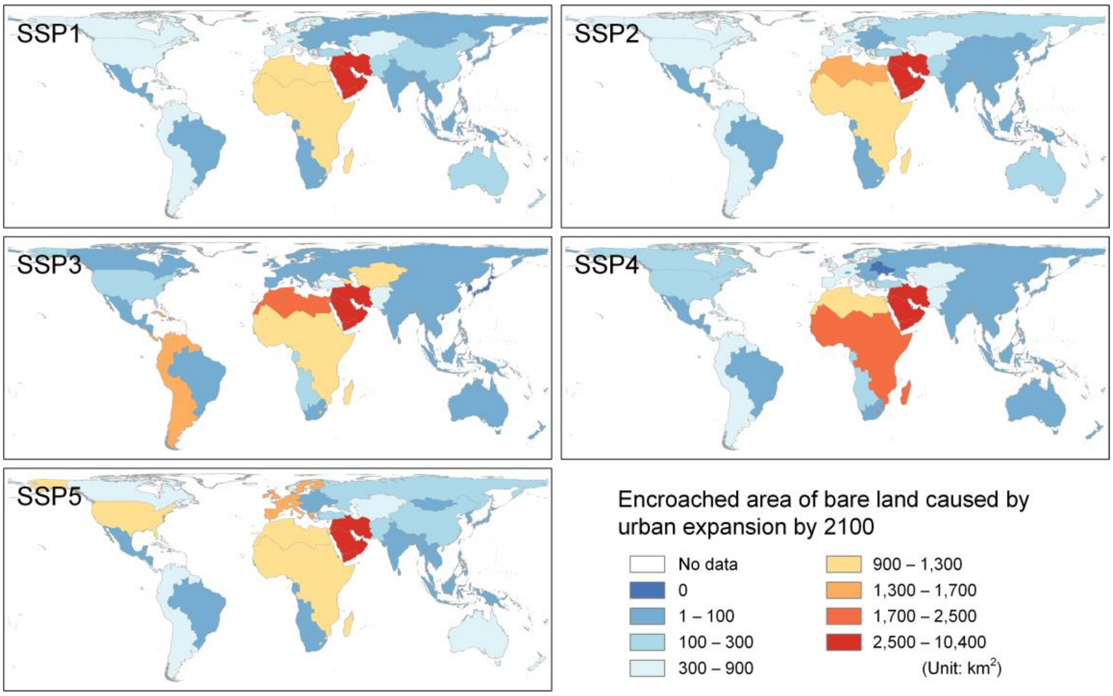

Supplementary Figure 11 | Encroached bare land area caused by urban expansion in each SSP scenario by 2100.

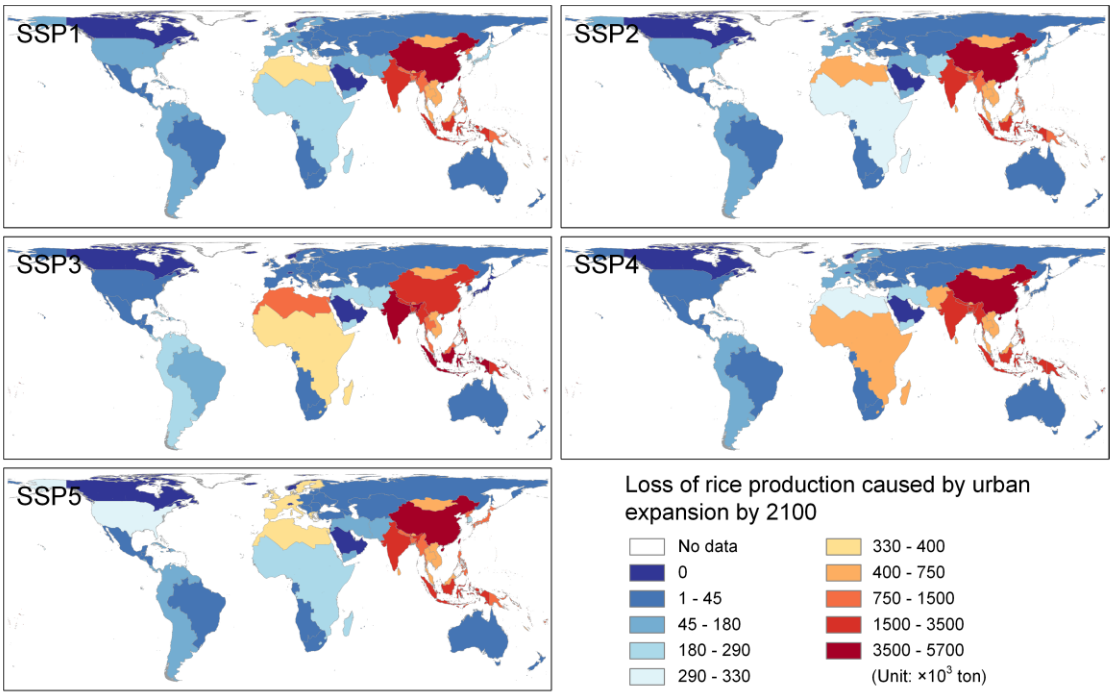

Supplementary Figure 12 | Loss of rice production caused by urban expansion in each SSP scenario by 2100.

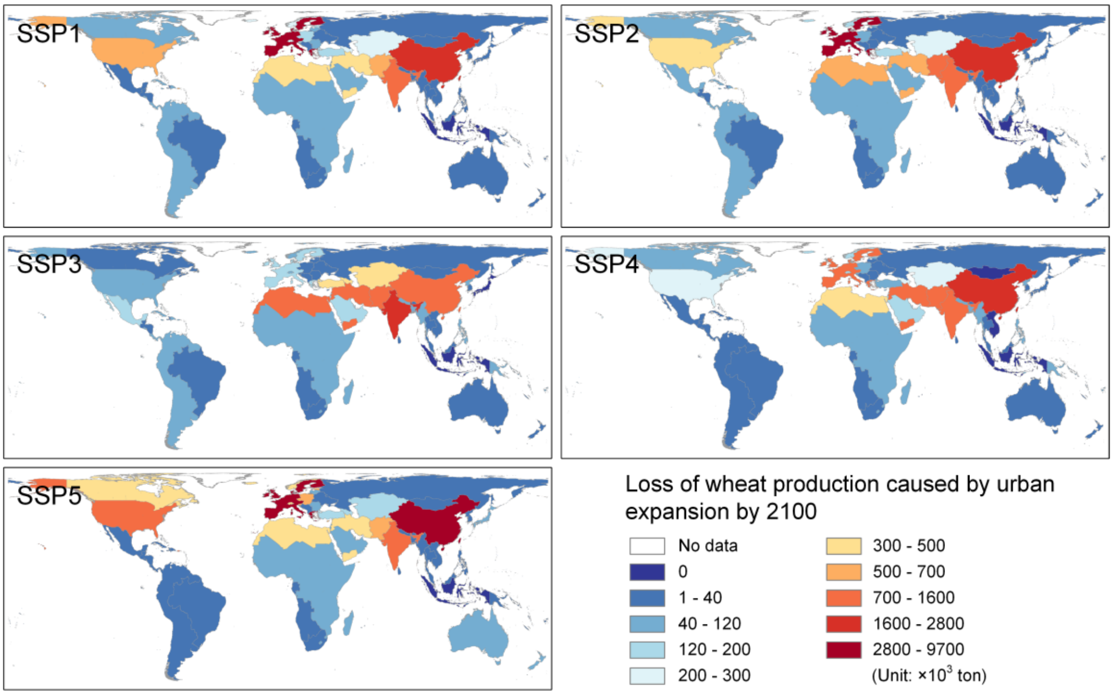

Supplementary Figure 13 | Loss of wheat production caused by urban expansion in each SSP scenario by 2100.

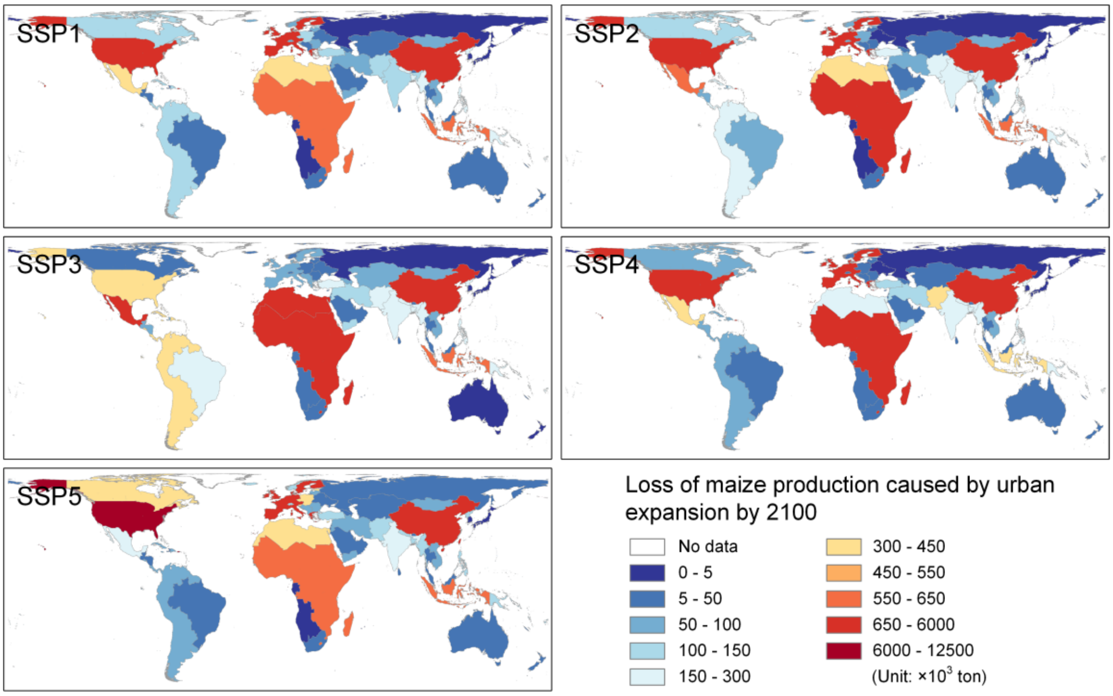

Supplementary Figure 14 | Loss of maize production caused by urban expansion in each SSP scenario by 2100.

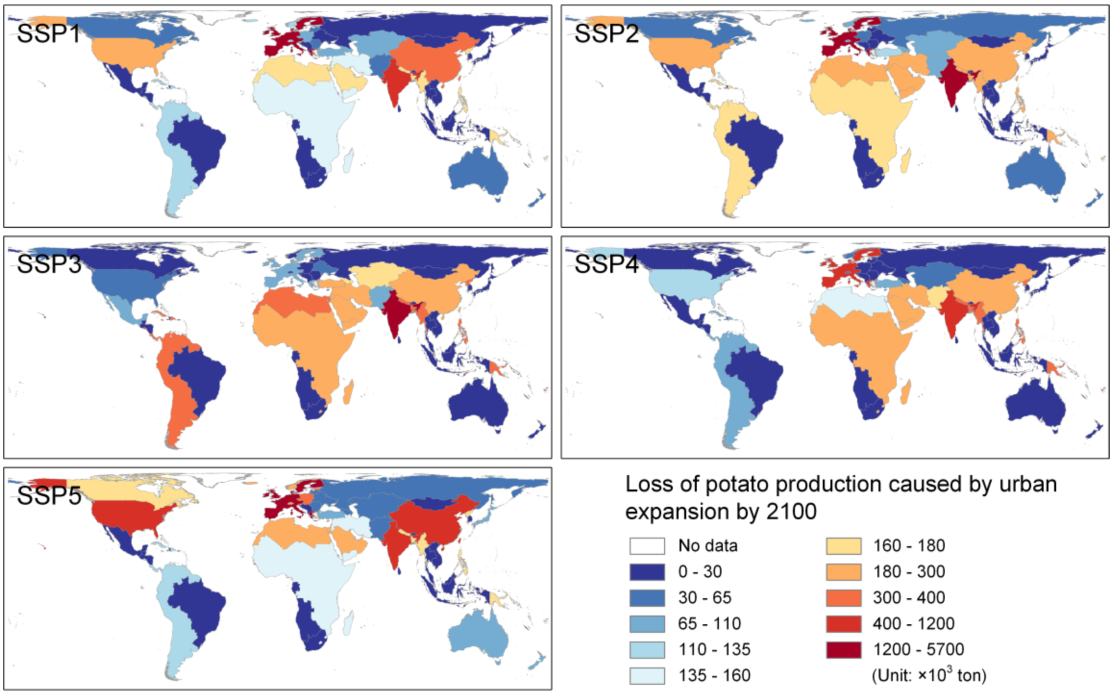

Supplementary Figure 15 | Loss of potato production caused by urban expansion in each SSP scenario by 2100.

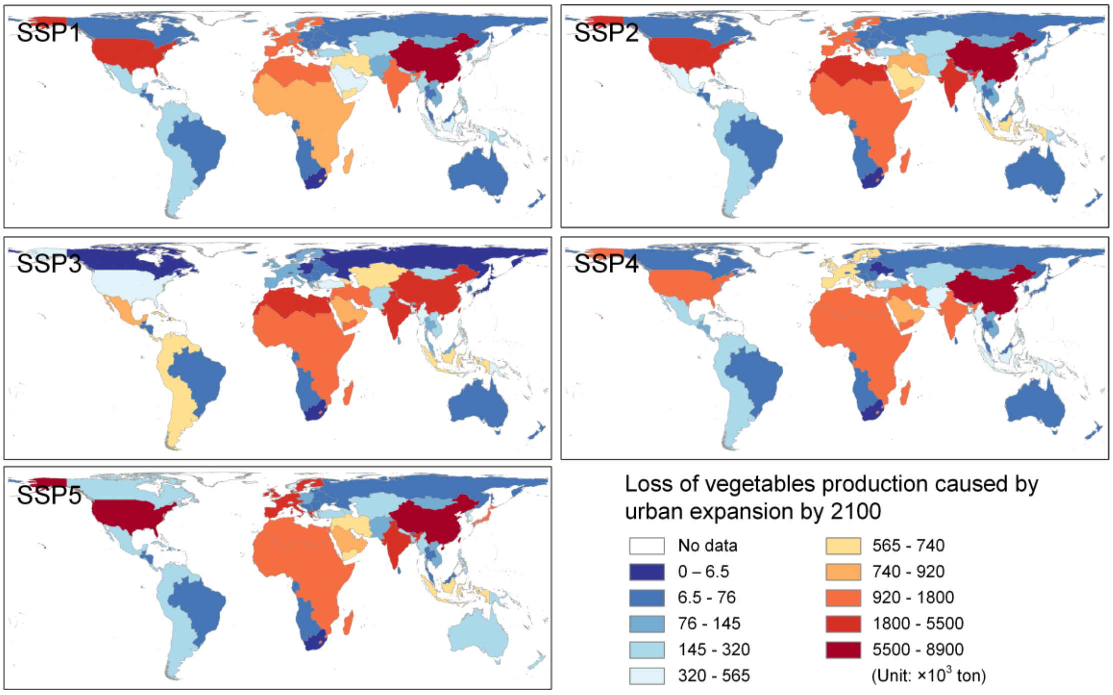

Supplementary Figure 16 | Loss of vegetables production caused by urban expansion in each SSP scenario by 2100.

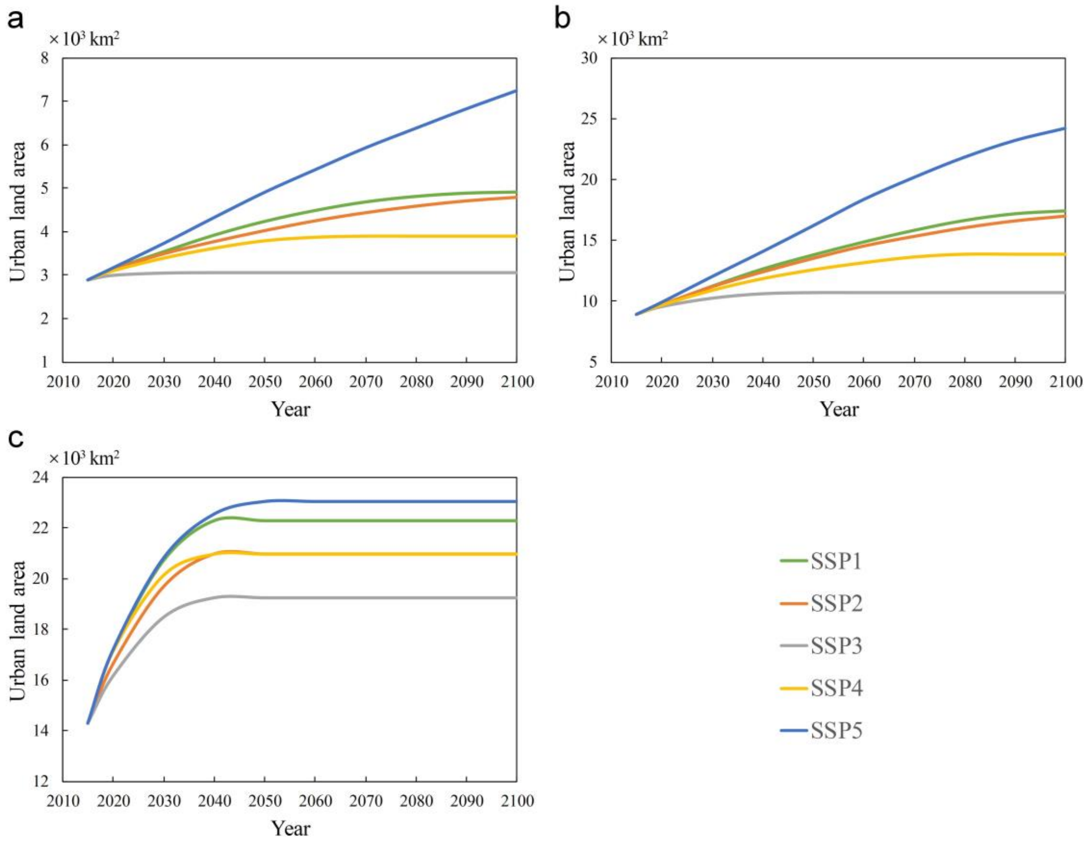

Supplementary Figure 17 | Urban area growth in the three major metropolitan areas from 2015 to 2100. (a) London metropolitan; (b) New York metropolitan; (c) Yangtze River Delta metropolitan.

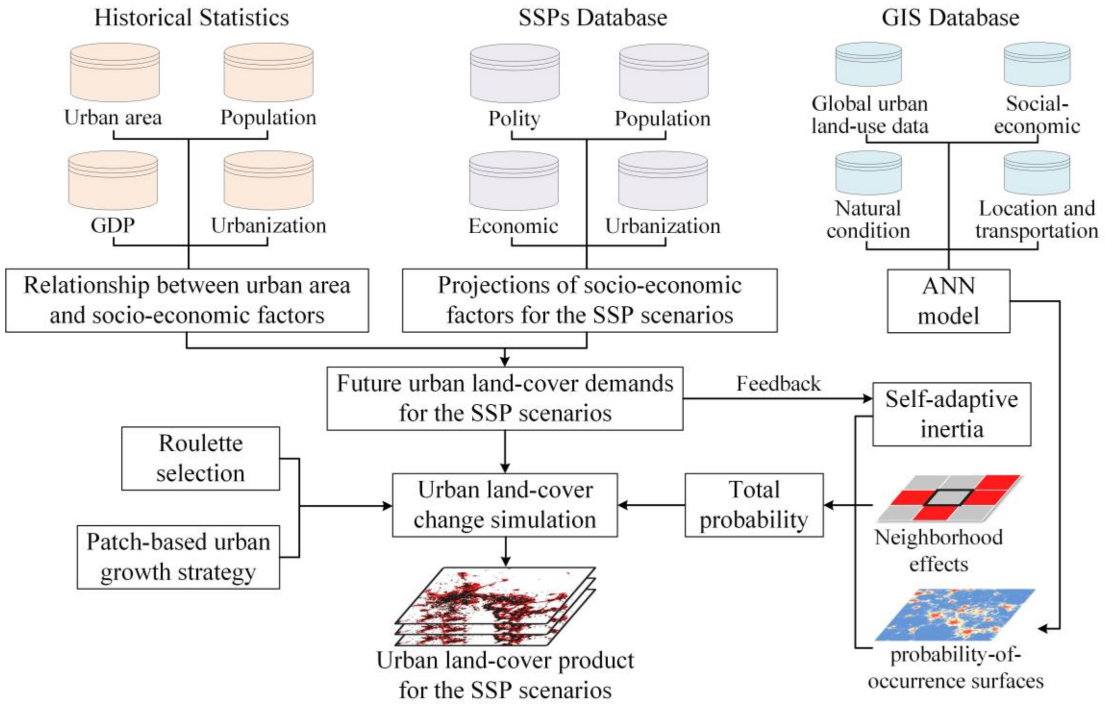

Supplementary Figure 18 | Flowchart of urban land cover simulation based on the SSP scenarios using the FLUS model.

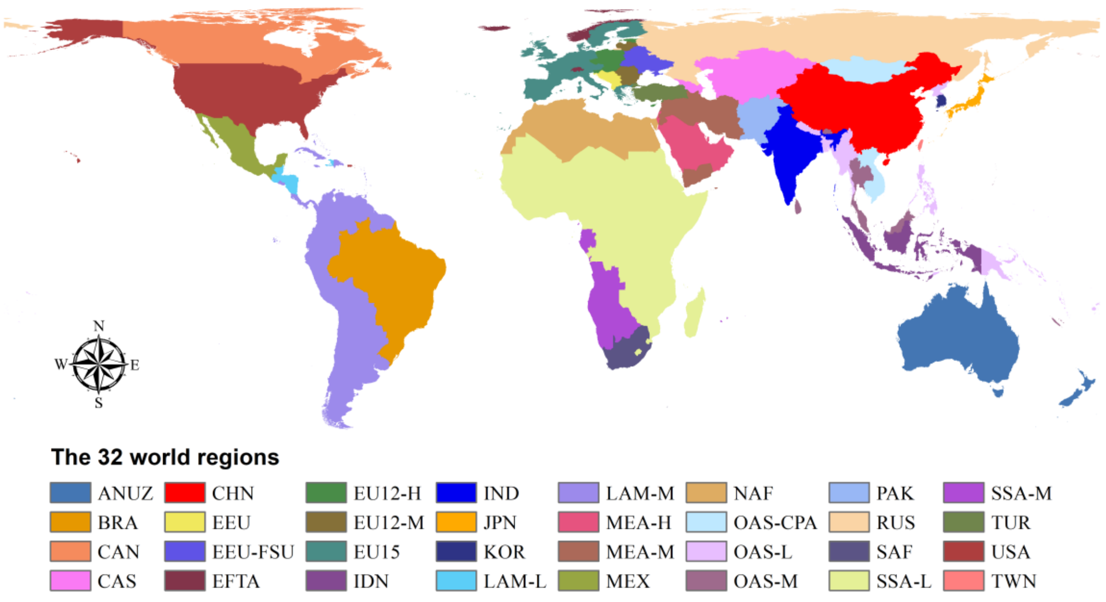

Supplementary Figure 19 | The 32 regions analysed by the SSPs around the world. The 32 regions are defined following the official SSP dataset (https://tntcat.iiasa.ac.at/SspDb).

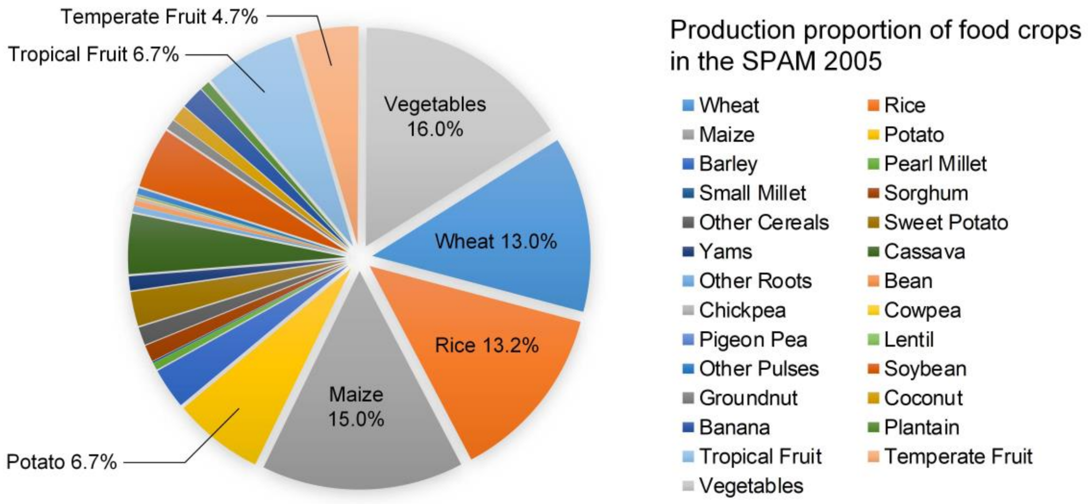

Supplementary Figure 20 | Production proportion of food crops in the SPAM 2005 map.

Supplementary Table 1 | The mean values of FoM (%) in the 32 regions for 100 global urban land cover simulations from 2000 to 2015

Region	FoM (%)		Contribution of the patch-based strategy
	Without Patch-based	Patch-based	
ANUZ	24.34	24.90	0.56
BRA	23.20	23.13	-0.07
CAN	20.36	20.71	0.35
CAS	24.41	24.36	-0.05
CHN	29.54	29.45	-0.09
EEU	16.96	16.76	-0.19
EEU-FSU	14.43	14.26	-0.18
EFTA	11.56	11.30	-0.26
EU12-H	16.76	16.65	-0.11
EU12-M	13.41	13.22	-0.19
EU15	14.86	14.82	-0.04
IDN	25.85	26.11	0.26
IND	31.01	30.91	-0.09
JPN	34.48	34.40	-0.09
KOR	28.38	28.62	0.24
LAM-L	22.42	22.95	0.53
LAM-M	26.28	26.22	-0.06
MEA-H	27.56	27.77	0.21
MEA-M	27.97	28.07	0.10
MEX	29.59	29.94	0.35
NAF	20.04	20.32	0.28
OAS-CPA	25.50	25.46	-0.04
OAS-L	22.54	22.52	-0.02
OAS-M	32.05	32.33	0.28
PAK	31.85	31.95	0.10
RUS	13.62	13.52	-0.10
SAF	26.57	26.55	-0.02
SSA-L	27.64	27.74	0.10
SSA-M	28.60	28.51	-0.09
TUR	26.95	27.15	0.20
TWN	35.99	36.24	0.25
USA	26.58	26.67	0.09

Note: Region names and acronyms follow the official SSP dataset (https://tntcat.iiasa.ac.at/SspDb).

Supplementary Table 2 | Simulated urban growth between 2015 and 2100 in each SSP

Region	Urban growth between 2015 and 2100 (km^2) / Growth rate since 2015 (%)				
	**SSP1**	**SSP2**	**SSP3**	**SSP4**	**SSP5**
**ANUZ**	14039(101.8)	13876(100.6)	3317(24.0)	9874(71.6)	32503(235.6)
**BRA**	4647(18.6)	6794(27.3)	14124(56.7)	3509(14.1)	5039(20.2)
**CAN**	10212(94)	9631(88.6)	1438(13.2)	5949(54.7)	26398(242.9)
**CAS**	4733(44.3)	5470(51.2)	10535(98.6)	4326(40.5)	4102(38.4)
**CHN**	33834(32.6)	28359(27.3)	21352(20.5)	27689(26.6)	38289(36.8)
**EEU**	882(28.5)	767(24.8)	909(29.3)	616(19.9)	1243(40.1)
**EEU-FSU**	938(3.8)	655(2.7)	2824(11.5)	414(1.7)	1831(7.5)
**EFTA**	6980(151.2)	6415(138.9)	1349(29.2)	4161(90.1)	15847(343.2)
**EU12-H**	3985(27.3)	2146(14.7)	343(2.4)	1296(8.9)	13535(92.8)
**EU12-M**	1108(11.3)	679(7.0)	1031(10.5)	726(7.4)	1254(12.8)
**EU15**	56291(65.6)	50391(58.7)	2970(3.5)	24699(28.8)	145923(170)
**IDN**	5679(36.5)	5997(38.6)	5921(38.1)	4233(27.2)	5939(38.2)
**IND**	28693(123.9)	33531(144.8)	39573(170.8)	25246(109)	33195(143.3)
**JPN**	775(3.2)	125(0.5)	0(0.0)	81(0.3)	5660(23.4)
**KOR**	326(9.9)	220(6.7)	102(3.1)	152(4.6)	1140(34.6)
**LAM-L**	1719(88.3)	2515(129.2)	4380(225.1)	5090(261.6)	1403(72.1)
**LAM-M**	8079(33.4)	12027(49.7)	25469(105.3)	7513(31.1)	7922(32.8)
**MEA-H**	8601(101.8)	12399(146.8)	15292(181.1)	15179(179.7)	13878(164.3)
**MEA-M**	10646(100.7)	15642(148)	24462(231.4)	26334(249.1)	12203(115.4)
**MEX**	4449(35.7)	7667(61.6)	18850(151.4)	3915(31.4)	3743(30.1)
**NAF**	4916(57.6)	6341(74.3)	10416(122.1)	4235(49.6)	4837(56.7)
**OAS-CP**	2084(54.4)	1967(51.3)	2493(65.0)	1855(48.4)	2026(52.9)
**OAS-L**	4557(81.2)	5107(91.0)	7630(136.0)	7936(141.5)	4445(79.2)
**OAS-M**	4187(50.9)	4726(57.4)	6320(76.8)	3307(40.2)	5097(61.9)
**PAK**	5988(151.7)	8874(224.8)	11507(291.5)	17638(446.8)	6116(154.9)
**RUS**	2317(7.6)	4557(15.0)	8235(27.1)	1514(5.0)	5652(18.6)
**SAF**	3159(43.8)	3238(44.9)	3776(52.4)	1664(23.1)	4233(58.7)
**SSA-L**	61762(262.1)	76787(325.9)	87343(370.7)	135336(574.4)	67784(287.7)
**SSA-M**	1647(75.8)	2102(96.8)	2754(126.8)	3226(148.5)	1741(80.1)
**TUR**	3583(49.6)	5416(74.9)	8350(115.5)	3173(43.9)	4567(63.2)
**USA**	106038(76.6)	97342(70.3)	17113(12.4)	54177(39.1)	262734(189.8)

**global**	406853(60.8)	431765(64.5)	360177(53.8)	405064(60.5)	740279(110.6)

Note: The 32 regions are defined following the official SSP dataset (https://tntcat.iiasa.ac.at/SspDb).

Supplementary Table 3 | The results of the panel data regression results for per capita urban land demand (unit: km^2 / million persons)

Coefficients	Estimate	Std. Error	t value	Pr(>|t|)
β_1(GDPC) / ($/person)	0.0006	0.00	2.96	<0.005
β_2(PU) / (%)	0.9154	0.24	3.84	<0.001
β_0(Intercept)				
ANUZ	182.80	20.99	8.71	<0.001
BRA	32.90	12.16	-12.33	<0.001
CAN	101.23	11.69	-6.98	<0.001
CAS	54.70	15.46	-8.28	<0.001
CHN	40.60	16.39	-8.68	<0.001
EEU	28.20	14.92	-10.36	<0.001
EEU-FSU	48.70	13.44	-9.98	<0.001
EFTA	-21.70	12.59	-16.24	<0.001
EU12-H	39.10	12.75	-11.27	<0.001
EU12-M	14.50	13.95	-12.06	<0.001
EU15	38.20	11.85	-12.20	<0.001
IDN	96.84	15.99	-5.38	<0.001
IND	28.20	17.54	-8.82	<0.001
JPN	84.64	11.61	-8.46	<0.001
KOR	18.80	12.08	-13.58	<0.001
LAM-L	14.70	14.73	-11.41	<0.001
LAM-M	19.40	12.28	-13.31	<0.001
MEA-H	54.90	11.71	-10.92	<0.001
MEA-M	19.20	13.48	-12.14	<0.001
MEX	35.80	12.23	-12.02	<0.001
NAF	55.80	14.25	-8.91	<0.001
OAS-CPA	64.50	18.26	-6.48	<0.001
OAS-L	33.20	17.36	-8.62	<0.001
OAS-M	45.10	15.71	-8.76	<0.001
PAK	28.50	16.79	-9.19	<0.001
RUS	41.90	13.05	-10.80	<0.001
SAF	103.21	13.49	-5.90	<0.001
SSA-L	28.30	17.45	-8.86	<0.001
SSA-M	47.40	15.50	-8.74	<0.001
TUR	15.00	13.06	-12.85	<0.001
USA	136.71	11.89	-3.88	<0.001

Supplementary Table 4 | The growth rate (%) of urban land area in the typical metropolitan areas and its regions by 2100

**Metropolitan area / Region**	**SSP1**	**SSP2**	**SSP3**	**SSP4**	**SSP5**
London	70.31	65.74	6.07	34.74	150.97
New York	95.68	90.35	19.83	55.49	171.19
Yangtze River Delta	55.98	46.81	34.68	46.79	61.39
EU-15	65.60	58.72	3.46	28.78	170.04
USA	76.61	70.33	12.36	39.14	189.81
China	32.55	27.29	20.54	26.64	36.84

Note: EU-15 represents the European Union member states that joined prior to 2004, including Austria, Belgium, Denmark, Finland, France, Germany, Greece, Ireland, Italy, Luxembourg, Netherlands, Portugal, Spain, Sweden, and the United Kingdom.

Supplementary Table 5 | Socio-economic and physical variables for estimating the probability-of-occurrence surfaces

**Spatial Variables**	**Year**	**Resolution**	**Data Sources**
Population	2010	0.5′	LandScan 2010 Global Population Project
GDP	2006	1 km	Ghosh et al. 2010 [1]
Human Influence Index	2004	0.5′	NASA Socioeconomic Data and Applications Center, Global Human Influence Index, v2
DEM	2000	0.5′	Hijmans et al. 2005 [2]
Slope	2000	0.5′	Retrieved from DEM
Distance to urban centers (population>30×10^3)	2014	1 km	United Nations, Department of Economic and Social Affairs, Population Division (2014).
Distance to main roads<br>Distance to ordinary roads	1980-2010	1 km	NASA, Socioeconomic Data and Applications Center, Global Roads Open Access Data Set (gROADS), v1
Distance to airports	2010	1 km	Huang et al. 2013 [3]
Distance to rivers and lakes	2015	1 km	ESA, Climate Research Data Package, CCI-LC maps, v2.0.7
Distance to sea	2015	1 km	
Ecoregions	2001	vector	World Wildlife Fund - Global 200 (terrestrial) Ecoregions, Olson et al. 2001 [4]

Note: A spatial resolution of 0.5′ is approximately 1 km at the equator. The eco-region data were resampled at 1 km for estimating the probability-of-occurrence surfaces.

Shared socioeconomic pathways (SSPs)

Recently, the SSPs have been adopted by the CMIP6, enabling researchers to conduct unified, comparable multi-scenario simulations and integrate such simulation products into climate change research[5-7]. The SSPs focus on key socio-economic factors, including demographic dynamics; economic development; technological change; social, cultural, and institutional changes; and policies[7]. In particular, unlike previous socio-economic scenarios, the SSPs divide the socio-economic challenges into two cateogires, ‘mitigation’ and ‘adaptation’, and set different scenarios accordingly[8]. To enable our research to better connect with existing climate research and facilitate future climate research, we use the latest recognized scenarios of the SSPs for simulating future urban land dynamics.

The five SSP scenarios are located at different sites in the ‘challenge space’ and are used to represent different socio-economic development pathways[9]. SSP1 is a people-oriented sustainable pathway that uses green roads[10]. In SSP1, by the end of this century, the global urbanization rate will reach 92%, with a rapidly growing GDP. SSP2 is a middle pathway between SSP1 and SSP3[11], and the urbanization rate and GDP values are also between the values corresponding to these two scenarios. SSP3 is a regional rivalry pathway contrary to global cooperation. In this scenario, as nationalism resurges, all countries focus only on domestic or, at most, regional issues. At the same time, in this scenario, economic development suffers the most, with a 60% urbanization rate, and the investments in education and technology decline[12]. SSP4 is

a divided pathway in which inequality and stratification increase both across and within countries. However, high-tech economic sectors are highly developed[13]; thus, in this scenario, economic development is relatively better than that in SSP3, and the global urbanization rate remains the same as that in SSP1. SSP5 is a fossil-fuelled development pathway, where people exploit abundant fossil fuel resources, the global economy grows at the highest speed, and the global urbanization rate reaches 92%. However, at the same time, people face severe mitigation challenges[14]. Notably, these only apply to the global situation. For the countries with different developments (such as developed countries, developing countries and low-income countries), their socio-economic assumptions in the same scenario are different or even the opposite. For example, developed countries have the fastest population growth in SSP5 and the slowest growth in SSP3, but low-income countries experience the opposite situation.

Future Land-Use Simulation (FLUS) model

The FLUS model[15] (http://www.geosimulation.cn/flus.html) assumes that the probability of a non-urban grid being converted into an urban grid (denoted as *TP*) is a product of the 'probability-of-occurrence' (*P*), neighbourhood effect (Ω), development restriction (*con*) and adjustment factor (*inertia*). Here the 'probability-of-occurrence' *P* refers to urban development potential, which is used to represent the site conditions of a grid for urban development. The 'probability-of-occurrence' is estimated using the ANN method and a set of variables

representing the driving factors of urban land expansion. The full list of these variables is shown in Supplementary Table 5. The neighbourhood effect Ω is used to represent the positive feedback in realistic urban development, i.e., non-urban grids are more likely to become urban if they have many neighbours of urban grids. The development restriction *con* is a binary variable with value 0 representing completely unsuitable condition for urban development or otherwise, 1. The adjustment factor (*inertia*) is used to adjust the growth rates of urban land in the simulations and facilitate convergence towards an expected quantity. The following text describe how these factors are calculated and prepared.

The estimation of the 'probability-of-occurrence' is based on the ANN method with a set of spatial variables. These spatial variables are selected mainly according to the findings of previous studies on urban land expansion simulation[16-18]. They include population, GDP, distance to the city centre, distance to the road network, distance to the airport, elevation, slope and the water resource condition, which collectively cover the major socioeconomic and natural factors affecting urban land expansion[16-18]. To take into account the ecological characteristics, we also acquire the ecoregion types from a world ecoregions map (Supplementary Table 5) and use them as nominal features in the ANN training. The corresponding data sources of all these spatial variables are provided in Supplementary Table 5. All these variables are processed to have a spatial resolution of 1 km with a spatial reference of *WGS_1984_Cylindrical_Equal_Area*.

The estimation of the 'probability-of-occurrence' is carried out separately for each of the 32 macro regions. Specifically, for each macro region, a sample is collected using a stratified random sampling approach with an equal proportion of 30% for urban and non-urban grids, respectively. This sample is used as a training dataset to train an ANN that yields the corresponding classification probabilities for 'urban' or 'non-urban'. The training procedure is based on the traditional back-propagation (BP) approach[19]. After the training procedure, for a non-urban grid i of land cover type k, its value of 'probability-of-occurrence' $P_{i,k}$ is represented using the 'urban' classification probability obtained from a trained ANN.

The neighbourhood effect for a non-urban grid i of land cover type k at time t, i.e., $\Omega_{i,k}^{t}$, is calculated as the fraction of existing urban grids in a 5×5 neighbourhood. The *inertia* factor is self-adaptive and adjusts the growth rates in the simulations based on the following equations:

$$Inertia_k^t = \begin{cases} Inertia_k^{t-1} & \text{if } \left|D_k^{t\text{-}1}\right| \leq \left|D_k^{t\text{-}2}\right| \\ Inertia_k^{t-1} \times \dfrac{D_k^{t\text{-}2}}{D_k^{t\text{-}1}} & \text{if } D_k^{t\text{-}1} < D_k^{t\text{-}2} < 0 \\ Inertia_k^{t-1} \times \dfrac{D_k^{t\text{-}1}}{D_k^{t\text{-}2}} & \text{if } 0 < D_k^{t\text{-}2} < D_k^{t\text{-}1} \end{cases} \quad (1)$$

where $Inertia_k^t$ is the inertia coefficient for land cover type k at time t. D_k^{t-1} represents the difference between the macro demand and allocated amount of the land cover type k until time t-1. The overall probability of land cover change for a grid is then estimated as follows:

$$TP_{i,k}^{t} = P_{i,k} \times \Omega_{i,k}^{t} \times inertia_k^t \times con_{c\rightarrow k} \quad (2)$$

where $TP_{i,k}^{t}$ is the overall probability of the grid cell i that changes into the land cover type k at time t; $P_{i,k}$ represents the probability-of-occurrence of land cover type k on grid cell i; $\Omega_{i,k}^{t}$ denotes the neighbourhood effect of land cover type k on grid cell i at time t; $inertia_{k}^{t}$ denotes the *inertia* value of land cover type k at time t; and $con_{c\rightarrow k}$ refers to a binary conversion constraint from the original land cover type c to the land cover type k (1 denotes possible conversion, and 0 denotes impossible conversion).

The simulation is based on a roulette selection mechanism, which is implemented with the following procedures: For a non-urban grid, its $TP_{i,k}^{t}$ (Equation (2)) is compared against a randomly generated value of [0, 1]. This non-urban grid is converted into an urban grid on the condition that $TP_{i,k}^{t}$ is greater than the random value. Otherwise, this non-urban grid remains unchanged.

The simulation of future urban land expansion is constrained by the projected urban land demand using the socioeconomic data provided by the SSP database. We use the socioeconomic data at year 2010, which is the starting year of the SSP database, to evaluate the performance of the model for urban land demand estimation. The results yield an overall error of 10.95% at the global level. To further reduce the impacts of this error on urban simulation, we implement the following adjustments:

$$\Delta A'_{r,2010\rightarrow t} = \frac{A'_{r,t}}{A'_{r,2010}} \quad (3)$$

$$A_{r,t} = A_{r,2010} \times \Delta A'_{r,2010\rightarrow t} \quad (4)$$

where $\Delta A'_{r,2010\rightarrow t}$ is the estimated growth rates in region r from year 2010 to

future year t; $A'_{r,2010}$ and $A'_{r,t}$ are the estimated urban land demand in region r at year 2010 and future year t; $A_{r,t}$ is the adjusted estimation of urban land demand in region r at future year t; $A_{r,2010}$ is the observed area of urban land cover at year 2010, which is derived from the actual global urban land map at year 2010. Therefore, the original estimations of urban land demand for future years are consistently adjusted using the reference of the actual area of urban land cover at year 2010. The adjusted estimation of urban land demand $A_{r,t}$ is then used to constrain the spatial simulation of urban land expansion.

Patch-based urban growth strategy

Rank-size distribution plays an important role and has been widely discussed in the urban geography literature. The distribution of population size for cities within a specific region has been found to follow the rank-size rule (Zipf's law)[20]. The distribution of urban land cover patches in a specific region has also been found to follow the rank-size rule[21, 22]. Zipf's law is the basic criterion we followed in the simulation by using a patch-based strategy, which is useful to yield better performances of urban simulation[23-25]. The estimation of the rank-size distribution exponent (λ) is usually performed through a linear regression on log–log plots, as the slope of the regression line is the estimate of the exponent[21]. Comparing the logarithms of patch rank and patch size, researchers have found that the exponents remain stable over time[21, 22]. This result indicates that larger patches tend to grow

relatively faster than smaller patches; thus, the exponents can be maintained in different periods. Therefore, we propose a patch-based urban growth strategy and apply it to the global urban simulation in this study, which can increase the potential for the development of large urban patches.

The core of the patch-based urban growth strategy is that larger patches will have a greater probability of development than smaller patches. Therefore, we propose a patch index to implement this strategy. Before starting each iteration, we count the distribution of all patch sizes in the region. When the iteration moves to a specific cell, we search for the urban-type cell in the neighbourhood and calculate the size of this patch. When there is a relatively larger urban patch near a cell, it has a larger patch index, which can reflect the enhanced effect of large cities and large patches on urban growth. Because larger cities generally have more resources, they have a greater scope for competitiveness and potential for development. This patch index is defined as:

$$PI_{i,U}^{t} = \begin{cases} 1 + \frac{\log\left(Patch_{i,U}^{t,max}\right)}{\log\left(Patch^{t,ave} + n \cdot Patch^{t,std}\right)}, & \frac{\log\left(Patch_{i,U}^{t,max}\right)}{\log\left(Patch^{t,ave} + n \cdot Patch^{t,std}\right)} \leq 1 \\ 2 & , \frac{\log\left(Patch_{i,U}^{t,max}\right)}{\log\left(Patch^{t,ave} + n \cdot Patch^{t,std}\right)} > 1 \end{cases} \quad (5)$$

where $PI_{i,U}^{t}$ represents the patch index of grid *i* for urban land cover type (*U*) at time *t*. $Patch_{i,U}^{t,max}$ represents the area of the largest urban patch (*U*) corresponding to the urban grid appearing in the neighbourhood of grid *i* at time *t*. $Patch^{t,ave}$ is the average area of all urban patches in the current region at time *t*. $Patch^{t,std}$ denotes the standard deviation in the area of all urban patches in the current region at time *t*. *n* is an adjustment parameter, which is set to 2 in this study. This adjustment factor can

reduce the overall impact of some extreme values. Therefore, in the patch-based strategy, $TP_{i,U}^{t}$ is multiplied by $PI_{i,U}^{t}$, resulting in $TP_{patch}{}_{i,U}^{t}$.

Figure of Merit (FoM)

Conventional accuracy indicators, such as the overall accuracy and Kappa coefficient, are used to evaluate the simulation performances of both changed and persistently non-changed simulations. This may lead to the overestimation of accuracies when evaluating the simulation performance[26]. However, FoM avoids overestimation by only focusing on the parts of land where changes have taken place[27, 28]. This index can be mathematically expressed as the ratio of the correct predicted change to the sum of the observed change and predicted change:

$$\mathrm{FoM} = \mathrm{B}/(\mathrm{A} + \mathrm{B} + \mathrm{C} + \mathrm{D}) \qquad (6)$$

where A represents the error area due to observed change predicted as persistence, B is the area of correctness due to observed change predicted as change, C denotes the area of error due to observed change predicted as change in the wrong category, and D denotes the area of error due to observed persistence predicted as change.

Model performance

We calibrate the model at the starting year (2000) using urban land-cover data from global land cover maps from the CCI-LC product. We then test and compare the performance of the model by simulating global urban land cover changes from 2000

to 2015 with/without the patch-based urban growth strategy. To ensure the reliability of the results, the model is run 100 times. In this way, we can compare the simulated results with the historical urban land-cover changes from the CCI-LC product to validate the model and determine the regions where the patch-based strategy plays a positive role.

We use FoM, which is an indicator ranging from 0% to 100% that reflects the simulation accuracy by focusing only on the part of the land that has changed, to measure the performance of the simulation results for urban land cover change from 2000 to 2015[27, 28]. Supplementary Table 1 shows the FoM values of the two methods for simulating global urban land cover change in 32 regions from 2000 to 2015. These methods, with and without the patch-based strategy, yield mean FoM values of 24.48% and 24.42% for the 32 regions, respectively. Our results are similar to those of other case studies on land cover change modelling. Previous comparative analysis has demonstrated that the common range of FoM values is between 10% and 30% for existing land cover change models[13, 20]. A recent global land cover simulation conducted by Li et al.[17] also reported similar results of FoM values, which ranged from 10% to 29%, with a mean value of 19%. Additionally, empirical studies have found that the value of FoM is strongly affected by the relative amount of observed net change[27]. Small FoM values were usually found in cases with a small amount of observed net change. For instance, Pontius et al.[27] revealed that the FoM value may decrease to less than 8% if the observed net change accounts for less than 2% of the

total land area. In our case, however, the observed urban land growth only accounts for 0.06% of the global terrestrial area, while the FoM values of our model exceed 24%. In this sense, the performance of our model is reliable, as indicated by the relatively higher FoM values, given a small amount of observed net change.

Based on the FoM values of the two methods (see Supplementary Table 1), we calculate the contribution of the patch-based urban growth strategy to the improvement in simulation accuracy. We find that the patch-based strategy can improve the simulation accuracy in many regions.

Comparison with other urbanization models

Existing global future urban land products are mainly based on the scenarios including single forecasting scenario[18, 29] and universal climate scenarios[30]. Models used for these projections include SLEUTH[25], URBANMOD (a modified version of the GEOMOD model)[18], and GGLM[30]. All these models except SLEUTH project future land changes based on a single set of initial land map.

In Zhou et al.'s research[29], they used SLEUTH model to simulate future land changes according to the historical trends captured from a series of historical land maps. However, their simulation only used a single scenario based on the historical trajectory. This simulation had not considered the potential pathways and uncertainties of future socio-economic factors and thus was incompatible with the recent IPCC framework. Moreover, the SLEUTH model can only input five spatial driving layers

(slope, excluded, urban, transportation, hillshade) for the urban land simulation, which corresponds to the five coefficients that control the behaviour of the system[25, 27]. These coefficients provide convenience for interpreting the mechanism of the model, but limit the consideration of more factors in the simulation process.

The URBANMOD model, which is a modified version of the GEOMOD model， was used by Seto et al. [18] to predict global urban land change by 2030. The single scenario formulated in their research is according to the projections of future socio-economic development made by the United Nations. In the original GEOMOD, new urban land is allocated to grids strictly following the order of suitability values from high to low, which is a deterministic approach[32]. Seto et al. [18] revised the original GEOMOD by allowing a certain degree of random perturbation in the allocation of new urban land.

The GGLM model[30], however, is for the simulation of global land use changes with multiple types (including urban land) under different future scenarios. The simulation is based on the downscaling of an existing future land product with a coarse resolution of 0.5 arc degree. The results of the GGLM model have a finer resolution of 1 km. The accuracies of the results depend largely on the original coarse-resolution future land product. In addition, in the GGLM model, the land type conversion of each grid is also determined by the land type with the highest conversion probability.

Our projections are generated using FLUS model coupled with the latest SSPs,

which describe potential pathways in the coming century concerning policy assumptions and the socio-economic narrative, being consistent with the recent IPCC framework. These projections thus provide better potential in supporting the research in related disciplines, such as ecological protection, water security, urban climate and global climate change.

Supplementary References

1. Ghosh, T. et al. Shedding Light on the Global Distribution of Economic Activity. The Open Geography Journal 3, 147-161 (2010).
2. Hijmans, R.J., Cameron, S.E., Parra, J.L., Jones, P.G. & Jarvis, A. Very high resolution interpolated climate surfaces for global land areas. INT J CLIMATOL 25, 1965-1978 (2005).
3. Huang, Z., Wu, X., Garcia, A.J., Fik, T.J. & Tatem, A.J. An open-access modeled passenger flow matrix for the global air network in 2010. PLOS ONE 8, e64317 (2013).
4. Olson, D.M. et al. Terrestrial Ecoregions of the World: A New Map of Life on Earth: A new global map of terrestrial ecoregions provides an innovative tool for conserving biodiversity. BIOSCIENCE 51, 933-938 (2001).
5. Kriegler, E. et al. A new scenario framework for climate change research: the concept of shared climate policy assumptions. CLIMATIC CHANGE 122, 401-414 (2014).
6. Kriegler, E. et al. The need for and use of socio-economic scenarios for climate change analysis: A new approach based on shared socio-economic pathways. Global Environmental Change 22, 807-822 (2012).
7. van Vuuren, D.P. et al. A new scenario framework for Climate Change Research: scenario matrix architecture. CLIMATIC CHANGE 122, 373-386 (2014).
8. O Neill, B.C. et al. A new scenario framework for climate change research: the concept of shared socioeconomic pathways. CLIMATIC CHANGE 122, 387-400 (2014).
9. O Neill, B.C. et al. The roads ahead: Narratives for shared socioeconomic pathways describing world futures in the 21st century. Global Environmental Change 42, 169-180 (2017).
10. van Vuuren, D.P. et al. Energy, land-use and greenhouse gas emissions trajectories under a green growth paradigm. Global Environmental Change 42, 237-250 (2017).
11. Fricko, O. et al. The marker quantification of the Shared Socioeconomic Pathway 2: A middle-of-the-road scenario for the 21st century. Global Environmental Change 42, 251-267 (2017).
12. Fujimori, S. et al. SSP3: AIM implementation of Shared Socioeconomic Pathways. Global Environmental Change 42, 268-283 (2017).
13. Calvin, K. et al. The SSP4: A world of deepening inequality. Global Environmental Change 42, 284-296 (2017).

14. Kriegler, E. et al. Fossil-fueled development (SSP5): An energy and resource intensive scenario for the 21st century. Global Environmental Change 42, 297-315 (2017).
15. Liu, X. et al. A future land use simulation model (FLUS) for simulating multiple land use scenarios by coupling human and natural effects. LANDSCAPE URBAN PLAN 168, 94-116 (2017).
16. Letourneau, A., Verburg, P.H. & Stehfest, E. A land-use systems approach to represent land-use dynamics at continental and global scales. ENVIRON MODELL SOFTW 33, 61-79 (2012).
17. Li, X. et al. A New Global Land-Use and Land-Cover Change Product at a 1-km Resolution for 2010 to 2100 Based on Human–Environment Interactions. ANN AM ASSOC GEOGR 107, 1040-1059 (2017).
18. Seto, K.C., Guneralp, B. & Hutyra, L.R. Global forecasts of urban expansion to 2030 and direct impacts on biodiversity and carbon pools. Proceedings of the National Academy of Sciences 109, 16083-16088 (2012).
19. Li, X. & Yeh, A.G. Neural-network-based cellular automata for simulating multiple land use changes using GIS. INT J GEOGR INF SCI 16, 323-343 (2002).
20. Rozenfeld, H.A.N.D. et al. Laws of population growth. Proceedings of the National Academy of Sciences 105, 18702-18707 (2008).
21. Fragkias, M. & Seto, K.C. Evolving rank-size distributions of intra-metropolitan urban clusters in South China. Computers, Environment and Urban Systems 33, 189-199 (2009).
22. Jiang, B., Yin, J. & Liu, Q. Zipf's law for all the natural cities around the world. INT J GEOGR INF SCI 29, 498-522 (2015).
23. Benguigui, L. & Blumenfeld-Lieberthal, E. A dynamic model for city size distribution beyond Zipf's law. Physica A: Statistical Mechanics and its Applications 384, 613-627 (2007).
24. Chen, Y., Li, X., Liu, X. & Ai, B. Modeling urban land-use dynamics in a fast developing city using the modified logistic cellular automaton with a patch-based simulation strategy. 28, 234-255 (2014).
25. Mansury, Y. & Gulyás, L. The emergence of Zipf's Law in a system of cities: An agent-based simulation approach. Journal of Economic Dynamics and Control 31, 2438-2460 (2007).
26. Chen, Y., Liu, X. & Li, X. Calibrating a Land Parcel Cellular Automaton (LP-CA) for urban growth simulation based on ensemble learning. INT J GEOGR INF SCI 31, 2480-2504 (2017).
27. Pontius Jr, R.G. et al. Comparing the input, output, and validation maps for several models of land change. The Annals of Regional Science 42, 11-37 (2008).
28. Pontius, R.G., Peethambaram, S. & Castella, J. Comparison of Three Maps at Multiple Resolutions: A Case Study of Land Change Simulation in Cho Don District, Vietnam. 101, 45-62 (2011).
29. Zhou, Y., Varquez, A.C.G. & Kanda, M. High-resolution global urban growth projection based on multiple applications of the SLEUTH urban growth model. SCI DATA 6 (2019).
30. Li, X. et al. A cellular automata downscaling based 1 km global land use datasets (2010-2100). SCI BULL 61, 1651-1661 (2016).
31. Clarke, K.C. & Gaydos, L.J. Loose-coupling a cellular automaton model and GIS: long-term urban growth prediction for San Francisco and Washington/Baltimore. INT J GEOGR INF SCI 12, 699-714 (1998).
32. Pontius Jr, R.G., Cornell, J.D. & Hall, C.A. Modeling the spatial pattern of land-use change with

GEOMOD2: application and validation for Costa Rica. Agriculture, Ecosystems \& Environment 85, 191-203 (2001).
